# Effect of Chrysin, a Flavonoid Present in Food, on the Skeletal System in Rats with Experimental Type 1 Diabetes

**DOI:** 10.3390/nu17020316

**Published:** 2025-01-16

**Authors:** Sylwia Klasik-Ciszewska, Piotr Londzin, Kacper Grzywnowicz, Weronika Borymska, Maria Zych, Ilona Kaczmarczyk-Żebrowska, Joanna Folwarczna

**Affiliations:** 1 Department of Pharmacology, Faculty of Pharmaceutical Sciences in Sosnowiec, Medical University of Silesia, 40-055 Katowice, Poland; sklasik-ciszewska@sum.edu.pl (S.K.-C.); piotr.londzin@vp.pl (P.L.); kacper.grzywnowicz@sum.edu.pl (K.G.); 2 Department of Pharmacognosy and Phytochemistry, Faculty of Pharmaceutical Sciences in Sosnowiec, Medical University of Silesia, 40-055 Katowice, Poland; weronika.borymska@sum.edu.pl (W.B.); mzych@sum.edu.pl (M.Z.); izebrowska@sum.edu.pl (I.K.-Ż.)

**Keywords:** experimental type 1 diabetes, chrysin, skeletal system, rats

## Abstract

Background: It seems that some substances of plant origin may exert health-promoting activities in diabetes and its complications, including those concerning bones. Chrysin (5,7-dihydroxyflavone), present in honey, some plants, and food of plant origin, has been reported to exert, among others, antioxidative, anti-inflammatory and antidiabetic effects. The aim of this study was to investigate the effects of chrysin on the skeletal system of rats with experimental type 1 diabetes (T1D). Methods: The experiments were carried out on mature male Wistar rats. T1D was induced by a single streptozotocin injection. Administration of chrysin (50 or 100 mg/kg p.o., once daily) began two weeks later and lasted four weeks. Serum bone turnover markers, bone mass, density and mineralization, mechanical properties and histomorphometric parameters of cancellous and compact bone were examined. Results: T1D profoundly affected bone metabolism, leading to worsening of bone strength in comparison with the healthy controls. After administration of chrysin, slight improvement of only some parameters was demonstrated in relation to the diabetic controls. Conclusions: Results of the present study indicate that chrysin may exert some very limited favorable effects on the skeletal system in diabetic conditions.

## 1. Introduction

It seems that some substances of plant origin, including flavonoids, may exert health-promoting activities in diabetes and its complications, including increased fracture risk. Chrysin (5,7-dihydroxyflavone) is a polyphenolic compound belonging to the flavonoid family. Chrysin’s two hydroxyl groups are both located on benzene ring A, and it has no oxygenation in ring B which distinguishes it from other flavonoids. The hydroxyl groups, and 2–3 carbon double bound and carbonyl group in position 4 (ring C), contribute to chrysin’s anti-inflammatory and antioxidant properties, shared with other flavonoids [1,2,3].

The first paper reporting on chrysin was published in 1893 [2]. Chrysin can be mainly found in products of bee origin such as honey and propolis. It occurs also in various plants belonging to different families: Geraniaceae (*Pelargonium crispum*), Passifloraceae (*Passiflora incarnata*), Bignoniaceae (*Oroxylum indicum*), Lamiaceae (*Scutellaria* spp.), Rubiaceae (*Morinda citrifolia*, *Hedyotis diffusa*), Zingiberaceae (*Alpinia oxyphylla*), Annonaceae (*Desmos cochinchinensis*), Asteraceae (*Centaurea omphalotricha*), Fabaceae (*Indigofera tinctoria*), Amaranthaceae (*Achyranthes aspera*). Moreover, chrysin is found in vegetables, fruits and mushrooms [2,3,4,5,6]. Commercially, chrysin is available as a dietary supplement [7].

Many pharmacological activities have been reported for chrysin: anticancer, antidiabetic, antiasthmatic, hepatoprotective, neuroprotective, nephroprotective and cardioprotective [1,3,4,5,8,9,10]. Chrysin has been reported to exert antiviral, antibacterial and antifungal activities [11,12]. Many of the demonstrated activities may result from chrysin’s antioxidant and anti-inflammatory properties [4,13,14,15,16]. There are also some inconsistent data; on the one hand, chrysin was reported to exert some estrogenic activity [17,18,19,20], and, on the other hand, to decrease estrogen production due to inhibition of aromatase (an enzyme converting androgens to estrogens) [2,21]. Limited experimental data from studies in vivo [17,22,23] and in vitro [24,25,26,27] indicate that chrysin may favorably affect the skeletal system.

Diabetes mellitus belongs to the most prevalent chronic diseases. Type 1 diabetes (T1D) is characterized by insulin deficiency due to autoimmune-mediated destruction of pancreatic β cells [28]. In type 2 diabetes, much more common, a non-autoimmune progressive loss of adequate insulin secretion is frequently based on the background of insulin resistance and metabolic syndrome [28]. One of numerous diabetes complications is diabetic bone disease [29]. The mechanism development of osteoporotic changes in diabetes is very complex and involves the damaging effect of insulin deficiency, hyperglycemia and oxidative stress on bone cells, leading to increased fracture rate in both type 1 and type 2 diabetes [30,31,32,33].

Taking into account potential antidiabetic [4,8,34,35] and antiosteoporotic [13] effects of chrysin, its favorable effect on the skeletal system in diabetes might be expected. So far, effects of chrysin on the skeletal system in diabetic conditions were not investigated in vivo. However, recently chrysin was reported to counteract hyperglycemia-induced bone marrow-derived mesenchymal stromal cells (BMSCs) dysfunction, promoting their osteogenic differentiation [36].

The aim of the present study was to determine the effect of the chrysin on the skeletal system in rats with experimental T1D induced by streptozotocin.

## 2. Materials and Methods

### 2.1. Substances Administered to Rats

Tested substance: chrysin (Sigma-Aldrich, St. Louis, MO, USA). Drugs used: streptozotocin (STZ; Cayman Chemical, Ann Arbor, MI, USA); ketamine (Ketamina 10%, Biowet Puławy Sp. z o. o., Puławy, Poland); xylazine (Xylapan, Vetoquinol Biowet, Gorzów Wlkp, Poland).

### 2.2. Animals

The biological material used in the present study was obtained during the experiment carried out on sexually mature (three-month-old at the start) male Wistar rats purchased from the Center of Experimental Medicine, Medical University of Silesia, Katowice, Poland. The animals were fed a standard laboratory diet (Labofeed B, Wytwórnia Pasz “Morawski”, Kcynia, Poland) ad libitum and had unrestricted access to water during the experiment. The diet contains 0.95% of calcium and 0.65% of phosphorus. The rats were kept in standard plastic cages (4–5 rats per cage) under a 12 h of light and 12 h of dark cycle; light turned on at 7:00 a.m.). The study protocol was in compliance with the 3R principles. All procedures of the experiments on animals were approved by the Local Ethics Committee, Katowice, Poland (approval numbers: 36/2015 and 114/2015).

### 2.3. Induction of Experimental Type 1 Diabetes Mellitus

Diabetes was induced by a single intraperitoneal injection of STZ (60 mg/kg) dissolved in 0.1 M citrate buffer (pH 4.5). STZ solution was prepared freshly before injections by dissolving 60 mg of STZ in 1 mL of citrate buffer. Two weeks after STZ injection, the non-fasting glucose level in the blood obtained from the tail vessels was measured with the use of a MicroDot glucometer equipped with test strips (Cambridge Sensor USA, Plainfield, IL, USA). If the blood glucose level exceeded 200 mg/dL, the animals were classified as diabetic and subjected to further steps of this study.

The rats were divided into four groups (*n* = 7–9):

1. Control—healthy control rats; *n* = 9.
2. T1D—control rats with STZ-induced diabetes; *n* = 8.
3. T1D + CH50—diabetic rats receiving chrysin (50 mg/kg); *n* = 9.
4. T1D + CH100—diabetic rats receiving chrysin (100 mg/kg); *n* = 7.

The rats were weighed once a week. Details concerning the in vivo experiment were already reported in our previous study [6]. This study shares control groups with our other study [37].

### 2.4. Chrysin Administration

Administration of chrysin started two weeks after the injections of STZ. Chrysin, suspended in water, was administered once daily via intragastric tube (p.o. – per os) for 28 days. Chrysin suspensions were prepared daily, before the administration, by suspending chrysin in the proportion of 50 or 100 mg of chrysin in 1 mL of water. Chrysin suspension was administered in a volume of 1 mL/kg. The healthy and diabetic control rats were administered the vehicle (water) in the same volume by the same route.

### 2.5. Biological Material Collection

The next day after administration of the last dose of chrysin, the rats were intraperitoneally injected with mixture of ketamine and xylazine in order to induce general anesthesia, and sacrificed by cardiac exsanguination. Bones (right and left femurs, left tibias and L4 vertebra) were isolated and frozen. The blood serum was stored frozen until the biochemical tests were performed.

### 2.6. Bone Macrometric Parameters

After isolation, the left tibia, left femurs and L4 vertebra were weighed using analytical balance Adventurer Pro type AV264CM (Ohaus Europe GmbH, Greifensee, Switzerland), and their length and mid-length diameter were determined using a digital caliper VOREL 15240 (Toya, Wrocław, Poland).

### 2.7. Biochemical Studies

The serum levels of non-fasting glucose and fructosamine were evaluated with BioSystems kits (BioSystems S.A., Barcelona, Spain). Insulin concentrations were measured using a Mercodia Ultrasensitive Rat Insulin ELISA kit (Mercodia AB, Uppsala, Sweden) [6]. The serum concentrations of the bone resorption marker—C-terminal telopeptide fragments of type I collagen (CTX-I; RatLaps) and the bone formation marker—osteocalcin were determined using ELISA kits (Immunodiagnostic Systems Ltd., Boldon, UK). The serum levels of calcium and inorganic phosphorus were determined with Pointe Scientific kits (Canton, MI, USA). All measurements were conducted using a Tecan Infinite M200 Pro plate reader with Magellan 7.2 software (Tecan Austria, Grödig, Austria).

### 2.8. Bone Composition and Mineralization Studies

To determine the dehydrated mass of the left femur, left tibia deprived of the proximal epiphysis and L4 vertebra, the bones were lyophilized at the temperature −53 °C and pressure of 0.03 mBa in a lyophilizer FreeZone 6 (Labconco, Kansas City, MO, USA). The lyophilized bones were weighed and then mineralized (ashed) to remove organic components from bones at a temperature of 640 °C for 48 h in a muffle furnace L9/11/C6 (Nabertherm, Lilienthal, Germany). The mineralized bones were weighed to determine the bone mineral mass. The bone water mass was calculated by subtracting the bone mass after lyophilization from the bone mass. The bone organic substance mass was determined by subtracting the mass of bone mineral from the bone mass after lyophilization. The ratio of the mass of bone mineral to the bone mass, the ratio of the mass of bone organic substances to the bone mass and the ratio of the mass of bone water to the bone mass were calculated to determine the bone content of mineral substances, organic substances and water, respectively. Bone density was determined employing Archimedes’ principle, with the use of an analytical balance Adventurer Pro type AV264CM with the density determination kit (Ohaus Europe GmbH, Greifensee, Switzerland). Bone mineral density was computed as the ratio of bone mineral mass to bone volume.

The ashed bones were dissolved in 6 M hydrochloric acid and then diluted in deionized water for the measurement of the content of calcium and phosphorus in the bone mineral. To determine calcium and phosphorus concentration, a spectrophotometric method was employed, with the use of an automated biochemical analyzer (BS-240, Mindray, Shenzhen, China) and Pointe Scientific kits (Canton, MI, USA).

### 2.9. Bone Histomorphometric Studies

The histological preparations were prepared as previously described from the right femurs [38,39]. In order to assess bone histomorphometric parameters, an Axio Imager.A1 microscope (Carl Zeiss, Göttingen, Germany) with Olympus DP71 camera (Olympus, Tokyo, Japan) and OsteoMeasure XP 1.3.0.1 software (OsteoMetrics, Decatur, GA, USA) were used. Results of histomorphometric measurements were presented according to the standardized nomenclature of the American Society for Bone and Mineral Research (ASBMR) [40].

In decalcified, stained with hematoxylin and eosin preparations of a longitudinal section of the distal femur (cancellous bone), the following parameters were measured in the metaphysis: bone volume/tissue volume ratio (BV/TV), trabecular thickness (Tb.Th), trabecular number (Tb.N) and trabecular separation (Tb.Sp). In nondecalcified preparations of cortical bone (femoral diaphysis; compact bone), the following histomorphometric parameters were determined: transverse cross-sectional area of the cortical bone (Ct.Ar), transverse cross-sectional area of the marrow cavity (Ma.Ar), transverse cross-sectional area of the total diaphysis (Tt.Ar), and transverse cross-sectional area of the marrow cavity/total diaphysis area ratio (Ma.Ar/Tt.Ar).

### 2.10. Bone Mechanical Property Studies

Mechanical properties of the left proximal tibial metaphysis and the left femoral diaphysis were performed in three-point bending tests using an Instron 3342 500N apparatus (Instron, Norwood, MA, USA) and Bluehill 2 version 2.14 software for data analysis, as previously described [39,41,42,43]. The load was applied perpendicularly to the long axis of the bone, in the mid-length of the femur (the distance between supporting points was 16 mm) or 3 mm from the edge of the proximal metaphysis in the tibia deprived of the proximal epiphysis, at the rate of 0.01 mm/s. The following parameters were determined: the Young’s modulus, load, displacement, energy, and stress for the yield point (0.05% offset), maximum load point, and fracture point. In order to determine moment of inertia in the break-section, necessary to calculate the values of Young’s modulus and stress, it was assumed that the femoral diaphysis was an elliptical pipe, and the proximal tibia metaphysis, a circular beam [39]. Mechanical properties of the right femoral neck were determined in a compression test, as previously described, using the same apparatus and software; the maximum load was measured [39,41].

### 2.11. Statistical Analysis

The results were presented as means ± SEM (standard error of the mean). Statistical analysis was performed using the Statistica 13.3 program (Tibco Software Inc., Palo Alto, CA, USA). One-way analysis of variance (ANOVA) followed by Fisher’s LSD (least significant difference) test was used for evaluation of the significance of the results. Results obtained in all groups of diabetic rats were compared with the healthy control rats, and results obtained in chrysin-treated diabetic rats were compared with the diabetic control rats. The results were considered statistically significant if *p* < 0.05.

## 3. Results

### 3.1. Effect of Chrysin on Body Mass and Concentration of Biochemical Markers of Carbohydrate Metabolism and Bone Turnover in the Serum in Rats with Experimental T1D

**Figure 1:**
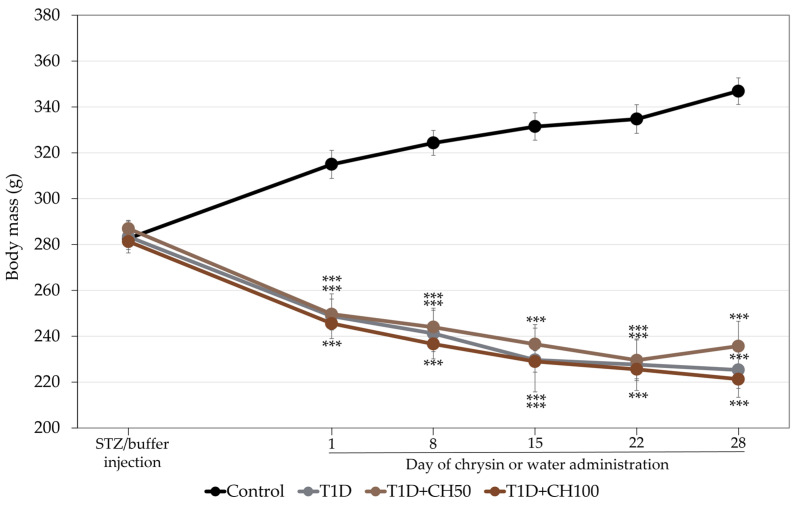
Effects of chrysin administered for 4 weeks on body mass in rats with experimental T1D. The results are presented as means ± standard error of the mean (SEM). Control—healthy control rats; T1D—diabetic control rats; T1D + CH50—diabetic rats treated with chrysin 50 mg/kg p.o. for 4 weeks; T1D + CH100—diabetic rats treated with chrysin 100 mg/kg p.o. for 4 weeks. One-way analysis of variance (ANOVA) followed by Fisher’s LSD test was used for evaluation of the significance of the results. *** *p* < 0.001—in comparison with the healthy control rats.

As previously reported [6], administration of STZ induced diabetes with a decrease in the serum concentration of insulin and increases in the serum concentrations of glucose and fructosamine in relation to the healthy control rats (Table 1). The body mass of the diabetic rats decreased (Figure 1). Administration of chrysin did not affect the biochemical parameters of carbohydrate metabolism and the body mass in diabetic rats.

In the diabetic rats (control and chrysin-treated), the serum calcium concentration was increased in relation to the healthy controls; there was not a significant effect of diabetes on the serum phosphorus concentration (Table 1). There was no effect of chrysin on the serum levels of calcium and phosphorus in the diabetic rats.

**Figure 2:**
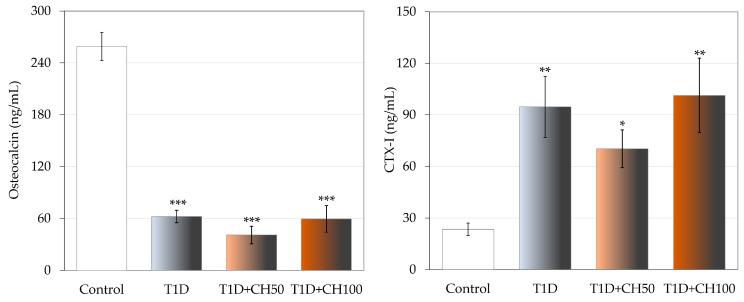
Effects of chrysin administered for 4 weeks on the serum bone turnover marker concentration in rats with experimental T1D. The results are presented as means ± standard error of the mean (SEM). Control—healthy control rats; T1D—diabetic control rats; T1D + CH50—diabetic rats treated with chrysin 50 mg/kg p.o. for 4 weeks; T1D + CH100—diabetic rats treated with chrysin 100 mg/kg p.o. for 4 weeks. CTX-I—C-terminal type I collagen fragments. One-way analysis of variance (ANOVA) followed by Fisher’s LSD test was used for evaluation of the significance of the results. * *p* < 0.05, ** *p* < 0.01, *** *p* < 0.001—in comparison with the healthy control rats.

Development of T1D induced by STZ significantly changed the bone turnover marker levels in diabetic rats in comparison with the healthy control rats (Figure 2). The serum concentration of osteocalcin strongly decreased and that of CTX-1 increased in the diabetic control rats. Administration of chrysin did not result in the improvement of the bone turnover marker levels, which had worsened due to diabetes.

### 3.2. Effect of Chrysin on the Bone Macrometric Parameters, Mass, Density and Mineralization in Rats with Experimental T1D

T1D resulted in statistically significant decreases in the femoral mass, length and diameter as well as density and mineral density, and phosphorus content in comparison with the healthy control rats (Table 2). Similar effects were observed in the tibia deprived of the proximal epiphysis (in which bone mineral mass/bone mass ratio also decreased) (Table 3) and L4 vertebra (Table 4). Treatment with chrysin did not affect most of the investigated parameters in comparison to the diabetic control rats, except for increases in some parameters concerning bone mineralization. In the tibia deprived of the proximal epiphysis, bone mineral density and the ratio of bone mineral mass to bone mass increased after chrysin administration at the lower dose (Table 3), and increases in bone mineral density and bone density in the L4 vertebra were observed after administration of chrysin at the higher dose (Table 4).

### 3.3. Effect of Chrysin on the Histomorphometric Parameters of the Femur in Rats with Experimental T1D

Diabetes decreased Tt.Ar and Ct.Ar, not affecting Ma.Ar and Ma.Ar/Tt.Ar in the femoral diaphysis in comparison with the healthy control rats, indicating a decrease in diaphyseal transverse growth (Table 5). Administration of chrysin did not affect the diabetes-induced changes in histomorphometric parameters of compact bone in diabetic rats.

Diabetic rats had insignificantly decreased values of BV/TV and Tb.Th in relation to the healthy controls. Administration of chrysin did not affect the investigated histomorphometric parameters of cancellous bone in diabetic rats (Table 6).

### 3.4. Effect of Chrysin on Bone Mechanical Properties of the Tibia and Femur in Rats with Experimental T1D

**Figure 3:**
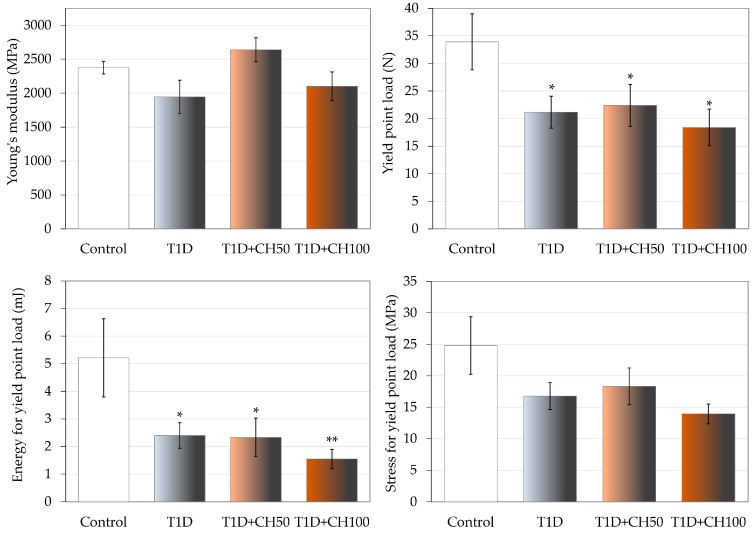
Effects of chrysin administered for 4 weeks on mechanical properties of the proximal tibial metaphysis in rats with experimental T1D. The results are presented as means ± standard error of the mean (SEM). Control—healthy control rats; T1D—diabetic control rats; T1D + CH50—diabetic rats treated with chrysin 50 mg/kg p.o. for 4 weeks; T1D + CH100—diabetic rats treated with chrysin 100 mg/kg p.o. for 4 weeks. One-way analysis of variance (ANOVA) followed by Fisher’s LSD test was used for evaluation of the significance of the results. * *p* < 0.05, ** *p* < 0.01—in comparison with the healthy control rats.

Development of T1D unfavorably affected the mechanical properties of the bones compared to the control rats. The diabetes-induced changes in cancellous bone of the proximal tibial metaphysis were very strong (Figure 3, Table 7). The values of load, displacement, energy and stress in the maximum load point and fracture point were significantly decreased. Also, the values of yield point load and energy decreased. Administration of chrysin at both doses did not significantly affect the mechanical properties of cancellous bone. Only a tendency to increase Young’s modulus in relation to the diabetic controls (*p* < 0.1) was demonstrated after administration of chrysin at a dose of 50 mg/kg p.o. (Figure 3).

**Figure 4:**
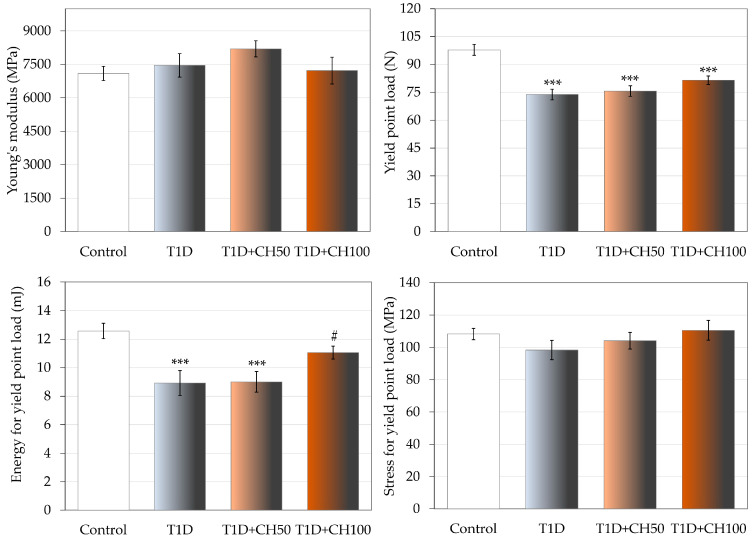
Effects of chrysin administered for 4 weeks on mechanical properties of the femoral diaphysis in rats with experimental T1D. The results are presented as means ± standard error of the mean (SEM). Control—healthy control rats; T1D—diabetic control rats; T1D + CH50—diabetic rats treated with chrysin 50 mg/kg p.o. for 4 weeks; T1D + CH100—diabetic rats treated with chrysin 100 mg/kg p.o. for 4 weeks. One-way analysis of variance (ANOVA) followed by Fisher’s LSD test was used for evaluation of the significance of the results. *** *p* < 0.001—in comparison with the healthy control rats. # *p* < 0.05—in comparison with the diabetic control rats (T1D group).

The changes induced by diabetes in compact bone of the femoral diaphysis were much weaker and limited to decreases in the values of load and energy in the yield point, and the load in maximum load and fracture points (Figure 4, Table 8). There was no effect of diabetes on the strength of the femoral neck. After administration of chrysin at a dose of 100 mg/kg, an increase in the value of energy for the yield point in the femoral diaphysis in comparison with the diabetic control rats was observed; there was also a tendency (*p* < 0.1) to increase the yield point load (Figure 4). Administration of chrysin did not affect other bone mechanical parameters in diabetic rats (Table 8).

## 4. Discussion

Patients with T1D suffer from bone disease as a complication of diabetes. The fracture risk is increased, especially that for hip fracture [29,31,44]. The pathophysiology of bone changes in diabetes is very complex and involves hyperglycemia, and consequent increase in advanced glycation end-products and their accumulation in bone matrix, leading to impaired bone cell function and bone quality. Insulin deficiency suppresses osteoblast function, and oxidative stress and the inflammatory environment contribute to development of bone disorders. Bone turnover in diabetic patients is decreased. Microvascular impairment may also contribute to diabetes-induced bone complications [29,33,45,46]. Nonetheless, fractures are an underappreciated complication of T1D, not always addressed in guidelines regarding diabetes [31]. Moreover, calculators of fracture risk used in clinical practice underestimate the risk in diabetes [44].

It may be hypothesized that beneficial effects on the skeletal system in diabetes may be achieved by using a diet containing natural compounds counteracting the pathological processes in the bone tissue. So the aim of this study was to investigate the effect of chrysin on the rat skeletal system in conditions of diabetes, with the emphasis on determination of bone mechanical properties.

The present study was carried out on sexually mature male Wistar rats with experimental T1D induced by STZ. The STZ-treated rats were highly hyperglycemic, with characteristic for diabetes hyperphagia, polydipsia and polyuria. STZ accumulates in and destroys pancreatic β cells, which results in insulin deficiency and development of hyperglycemia [47,48]. This model of experimental diabetes is widely used in experimental pharmacology as it mimics the changes occurring in the human body due to T1D, including damaging effects on the skeletal system [39,49].

Results of the present study confirmed a very strong damaging effect of the T1D model used on the skeletal system of male rats. The serum concentration of a bone formation marker, osteocalcin, decreased, and concentration of C-terminal telopeptide fragments of type I collagen increased, indicating increased bone resorption, as observed previously in experimental diabetes in rodents [30,39]. The serum calcium concentration was increased, probably due to increased bone resorption, consistently with our previous study [39]. T1D decreased bone mass, mineralization and macrometric parameters, worsened histomorphometric parameters and mechanical properties in both cancellous (the proximal tibial metaphysis) and compact (the femoral diaphysis) bone. The changes were similar to those observed in our previous studies performed on female rats with STZ-induced diabetes [49,50].

In the present study, chrysin was administered to diabetic rats at two, rather high, daily doses (50 mg/kg and 100 mg/kg p.o.). The doses were chosen based on literature data, taking into account its low bioavailability [9,51]. Chrysin was administered for 4 weeks; the period of administration corresponded to about 2.5 years in humans, taking into account the life-length of rats and humans [52]. Such a period was sufficient to demonstrate the effects of different substances of plant origin on the rat skeletal system [41,49,50].

As previously reported [6], administration of chrysin (50 or 100 mg/kg p.o.) did not affect the serum concentration of glucose in diabetic rats (the rats remained highly hyperglycemic). There was also no influence of chrysin on the serum fructosamine and insulin concentrations [6]. Most of the investigated parameters of the skeletal system of diabetic rats were not affected by chrysin administration, and there was a lack of effect on the serum concentrations of bone turnover markers. Nevertheless, some beneficial changes concerning bone mineralization in the vertebra and tibia (but not in the femur) were noted. The changes were not dose dependent; in the tibia, increases in the bone mineral density and bone content of mineral substances were observed after administration of chrysin at the lower dose, whereas in the vertebra, increases in the bone density and bone mineral density occurred after administration of chrysin at the higher dose. The improvement in those parameters did not lead to significant amelioration of the profound diabetes-induced changes in bone mechanical properties. Only the value of energy for the yield point in the femoral diaphysis (compact bone) increased significantly after administration of chrysin at the higher dose, and the value of the yield point load tended to increase in relation to the diabetic controls. In the proximal tibial metaphysis (cancellous bone), the value of Young’s modulus tended to increase after administration of chrysin at the lower dose, consistently with the improvement in bone mineralization. However, other mechanical parameters of cancellous bone remained strongly worsened in chrysin-treated diabetic rats. Summing up, the effects of chrysin on the skeletal system of diabetic rats were very limited.

Results of the present study differ from those of previous reports on experimental studies which demonstrated more significant favorable effects of chrysin in other models of bone loss [17,22,23]. In those models, chrysin was administered orally at the same doses as in present study (50 mg/kg and 100 mg/kg for 6 weeks) [17] and 100 mg/kg for 2 weeks [22,23]. None of the studies investigated bone mechanical properties. Contrary to the present results, in the rat model of postmenopausal osteoporosis (ovariectomy-induced estrogen deficiency), chrysin administration counteracted the changes in bone turnover markers, and increased bone mass and mineral mass [17]. In a model of bone loss induced by retinoic acid administration, chrysin counteracted the changes in the serum bone turnover markers, but not in bone mineral content and density, when administered for 2 weeks after the induction of osteoporotic changes [22]. Chrysin was also reported to counteract the development of retinoic acid-induced changes in bone mineral density and mineralization when administered with retinoic acid for 2 weeks [23]. Moreover, chrysin was reported to potentially exert a favorable effect on bone regeneration. Chrysin induced osteogenic differentiation in murine preosteoblast MC3T3-E1 cells [27] and human dental pulp stem cells in vitro [24]. Chrysin also increased proliferation of mouse mesenchymal stem cells and their differentiation to osteoblasts, when released from biocomposite scaffolds [25]. Also, local administration of chrysin on the calvarial defect area on which decalcified bone matrix scaffold was applied promoted bone regeneration in rats with STZ-induced diabetes [36]. One may speculate that the diabetes-induced changes in the skeletal system were too profound to be efficiently counteracted by chrysin. Taking into account the lack of effect of chrysin administration on the serum glucose concentration, the mechanisms of its effect on the skeletal system may be speculated based on its activities reported so far.

The slight favorable effects of chrysin, demonstrated in the present study, could be related to its antioxidant and anti-inflammatory properties. There are many reports on the antioxidant effects of chrysin [4,9,51]. The parameters of oxidative stress were not investigated in the present study. However, in lenses of rats whose bones were investigated in this study, chrysin decreased the activity of antioxidative enzymes, reduced the levels of the oxidative damage markers and improved the oxidative stress index [6]. It was previously reported that chrysin could protect bone marrow stem cells from high glucose-induced oxidative stress through activation of the PI3K/ATK/Nrf2 signaling pathway; moreover, chrysin-treated BMSCs displayed a higher proliferative rate [36]. Also, the anti-inflammatory effect of chrysin is well documented [15,22]. For example, chrysin significantly reduced the serum levels of pro-inflammatory cytokines, interleukin-1β (IL-1β), IL-6, TNF-α and RANTES (regulated on activation normal T cell expressed and secreted) chemokine in retinoic acid-treated rats [22].

Numerous flavonoids are classified as phytoestrogens (for example, genistein, formononetin, glabridin, liquiritigenin); they have been demonstrated to exert favorable effects on the skeletal system in different experimental models of bone loss [53,54,55,56,57]. The data on the effect of chrysin on estrogenic pathways are inconsistent. Chrysin was reported to exert estrogenic effects [18,19], but to have very low binding affinity to estrogen receptors [20]. The osteogenic effect of chrysin on preosteoblastic cells involved estrogenic receptors [27]. In an in silico study, chrysin exhibited estrogenic-like activity [17]. Estrogenic activity might have been responsible for the abovementioned effects on the development of osteoporosis in ovariectomized rats [17]. Estrogens and their receptors occur and are important also in male organisms. Estrogen deficiency plays primary role in hypogonadal bone loss in men [58]. Moreover, it was demonstrated that selective estrogen receptor modulators (SERM) reduce bone loss in men undergoing androgen deprivation therapy for prostate cancer [59]. Therefore, it may be assumed that the estrogenic effect of chrysin might have a beneficial effect on the skeletal system of male rats in the present study. However, the direct estrogenic effect of chrysin could be counteracted by the inhibitory effect of chrysin on aromatase activity and estrogen production [2,21].

## 5. Conclusions

In conclusion, the result of the present comprehensive study on the effects of chrysin on the skeletal system of male rats with experimental type 1 diabetes induced by STZ demonstrated only its very limited favorable effects on the investigated bone parameters, which were not dose dependent. The results do not support the hypothesis that chrysin may be a potential drug used in prophylaxis and treatment of diabetes-induced bone complications.

## Figures and Tables

**Table 1:** Effects of chrysin administered for 4 weeks on the serum biochemical parameters of carbohydrate and mineral metabolism in rats with experimental T1D.

Parameter/Group	Control	T1D	T1D + CH50	T1D + CH100
Glucose (mg/dL)	141.4 ± 11.0	641.8 ± 28.6 ***	687.9 ± 21.5 ***	588.8 ± 53.8 ***
Fructosamine (µmol/L)	281.9 ± 9.8	495.6 ± 23.1 ***	483.3 ± 32.5 ***	444.4 ± 23.7 ***
Insulin (µg/L)	0.434 ± 0.096	0.089 ± 0.019 ***	0.171 ± 0.045 **	0.136 ± 0.057 **
Calcium (mg/dL)	8.88 ± 0.18	10.16 ± 0.29 **	9.85 ± 0.21 **	10.18 ± 0.38 **
Phosphorus (mg/dL)	6.75 ± 0.19	8.19 ± 1.13	7.33 ± 0.51	7.85 ± 1.09

The results are presented as means ± standard error of the mean (SEM). Control—healthy control rats; T1D—diabetic control rats; T1D + CH50—diabetic rats treated with chrysin 50 mg/kg p.o. for 4 weeks; T1D + CH100—diabetic rats treated with chrysin 100 mg/kg p.o. for 4 weeks. One-way analysis of variance (ANOVA) followed by Fisher’s LSD test was used for evaluation of the significance of the results. ** *p* < 0.01, *** *p* < 0.001—in comparison with the healthy control rats. Results concerning carbohydrate metabolism were already published [6].

**Table 2:** Effects of chrysin administered for 4 weeks on the bone macrometric parameters, mass, density and mineralization in the femur in rats with experimental T1D.

Parameter/Group	Control	T1D	T1D + CH50	T1D + CH100
Bone length (mm)	36.40 ± 0.34	34.28 ± 0.31 ***	34.26 ± 0.23 ***	34.59 ± 0.22 ***
Bone diameter (mm)	3.60 ± 0.05	3.33 ± 0.09 *	3.39 ± 0.06 *	3.37 ± 0.10 *
Bone mass (g)	0.855 ± 0.022	0.702 ± 0.024 ***	0.693 ± 0.015 ***	0.717 ± 0.027 ***
Bone density (g/cm^3^)	1.626 ± 0.005	1.551 ± 0.012 ***	1.560 ± 0.014 ***	1.552 ± 0.013 ***
Bone mineral density (g/cm^3^)	0.753 ± 0.006	0.695 ± 0.010 ***	0.714 ± 0.011 **	0.709 ± 0.007 **
Bone mineral mass (g)	0.396 ± 0.009	0.314 ± 0.010 ***	0.318 ± 0.008 ***	0.327 ± 0.011 ***
Bone water mass (g)	0.259 ± 0.007	0.218 ± 0.010 **	0.212 ± 0.006 ***	0.220 ± 0.011 **
Bone organic substances mass (g)	0.201 ± 0.006	0.169 ± 0.007 ***	0.163 ± 0.004 ***	0.170 ± 0.006 ***
Bone mineral mass/bone mass ratio (g/g)	0.463 ± 0.003	0.448 ± 0.005	0.458 ± 0.006	0.457 ± 0.005
Bone water mass/bone mass ratio (g/g)	0.302 ± 0.003	0.310 ± 0.006	0.306 ± 0.006	0.306 ± 0.007
Bone organic substances mass/bone mass ratio	0.234 ± 0.002	0.241 ± 0.006	0.236 ± 0.002	0.237 ± 0.003
Calcium content (g/g bone mineral)	0.422 ± 0.003	0.419 ± 0.003	0.420 ± 0.003	0.424 ± 0.004
Phosphorus content (g/g bone mineral)	0.171 ± 0.001	0.167 ± 0.001 *	0.167 ± 0.001 *	0.167 ± 0.001 *

The results are presented as means ± standard error of the mean (SEM). Control—healthy control rats; T1D—diabetic control rats; T1D + CH50—diabetic rats treated with chrysin 50 mg/kg p.o. for 4 weeks; T1D + CH100—diabetic rats treated with chrysin 100 mg/kg p.o. for 4 weeks. One-way analysis of variance (ANOVA) followed by Fisher’s LSD test was used for evaluation of the significance of the results. * *p* < 0.05, ** *p* < 0.01, *** *p* < 0.001—in comparison with the healthy control rats.

**Table 3:** Effects of chrysin administered for 4 weeks on the bone macrometric parameters, mass, density and mineralization in the tibia deprived of the proximal epiphysis in rats with experimental T1D.

Parameter/Group	Control	T1D	T1D + CH50	T1D + CH100
Bone length ^&^ (mm)	40.17 ± 0.34	38.46 ± 0.37 ***	38.24 ± 0.27 ***	38.58 ± 0.22 **
Bone diameter ^&^ (mm)	2.88 ± 0.05	2.74 ± 0.05 *	2.69 ± 0.03 **	2.71 ± 0.07 *
Bone mass (g)	0.535 ± 0.012	0.438 ± 0.017 ***	0.425 ± 0.008 ***	0.440 ± 0.019 ***
Bone density (g/cm^3^)	1.636 ± 0.007	1.586 ± 0.009 ***	1.602 ± 0.009 **	1.593 ± 0.010 **
Bone mineral density (g/cm^3^)	0.776 ± 0.008	0.723 ± 0.008 ***	0.752 ± 0.009 *#	0.739 ± 0.007 **
Bone mineral mass (g)	0.253 ± 0.006	0.200 ± 0.007 ***	0.200 ± 0.004 ***	0.204 ± 0.008 ***
Bone water mass (g)	0.148 ± 0.004	0.129 ± 0.008 *	0.122 ± 0.004 **	0.132 ± 0.007
Bone organic substances mass (g)	0.133 ± 0.004	0.109 ± 0.005 ***	0.104 ± 0.002 ***	0.104 ± 0.004 ***
Bone mineral mass/ bone mass ratio	0.474 ± 0.004	0.456 ± 0.003 **	0.470 ± 0.005 #	0.464 ± 0.003
Bone water mass/ bone mass ratio	0.276 ± 0.005	0.294 ± 0.009	0.286 ± 0.007	0.300 ± 0.006
Bone organic substances mass/bone mass ratio	0.249 ± 0.003	0.250 ± 0.008	0.245 ± 0.003	0.236 ± 0.004
Calcium content (g/g bone mineral)	0.423 ± 0.003	0.429 ± 0.003	0.439 ± 0.007	0.431 ± 0.002
Phosphorus content (g/g bone mineral)	0.172 ± 0.002	0.170 ± 0.002	0.170 ± 0.002	0.169 ± 0.001

The results are presented as means ± standard error of the mean (SEM). Control—healthy control rats; T1D—diabetic control rats; T1D + CH50—diabetic rats treated with chrysin 50 mg/kg p.o. for 4 weeks; T1D + CH100—diabetic rats treated with chrysin 100 mg/kg p.o. for 4 weeks. ^&^—whole tibia. One-way analysis of variance (ANOVA) followed by Fisher’s LSD test was used for evaluation of the significance of the results. * *p* < 0.05, ** *p* < 0.01, *** *p* < 0.001—in comparison with the healthy control rats. # *p* < 0.05—in comparison with the diabetic control rats (T1D group).

**Table 4:** Effects of chrysin administered for 4 weeks on the bone macrometric parameters, mass, density and mineralization in the L4 vertebra in rats with experimental T1D.

Parameter/Group	Control	T1D	T1D + CH50	T1D + CH100
Bone mass (g)	0.208 ± 0.010	0.155 ± 0.005 ***	0.150 ± 0.004 ***	0.148 ± 0.012 ***
Bone density (g/cm^3^)	1.559 ± 0.013	1.472 ± 0.015 *	1.474 ± 0.016 *	1.547 ± 0.046 #
Bone mineral density (g/cm^3^)	0.703 ± 0.009	0.651 ± 0.019 *	0.672 ± 0.013	0.707 ± 0.019 #
Bone mineral mass (g)	0.094 ± 0.004	0.069 ± 0.003 ***	0.068 ± 0.002 ***	0.068 ± 0.006 ***
Bone water mass (g)	0.062 ± 0.004	0.048 ± 0.002 ***	0.045 ± 0.002 ***	0.044 ± 0.003 ***
Bone organic substances mass (g)	0.052 ± 0.002	0.038 ± 0.002 ***	0.037 ± 0.001 ***	0.036 ± 0.003 ***
Bone mineral mass/ bone mass ratio	0.451 ± 0.005	0.442 ± 0.010	0.456 ± 0.006	0.458 ± 0.006
Bone water mass/ bone mass ratio	0.300 ± 0.006	0.310 ± 0.013	0.298 ± 0.008	0.296 ± 0.009
Bone organic substances mass/bone mass ratio	0.249 ± 0.002	0.248 ± 0.004	0.246 ± 0.005	0.246 ± 0.003
Calcium content (g/g bone mineral)	0.436 ± 0.010	0.444 ± 0.008	0.447 ± 0.009	0.441 ± 0.008
Phosphorus content (g/g bone mineral)	0.171 ± 0.001	0.169 ± 0.002	0.171 ± 0.001	0.166 ± 0.001

The results are presented as means ± standard error of the mean (SEM). Control—healthy control rats; T1D—diabetic control rats; T1D + CH50—diabetic rats treated with chrysin 50 mg/kg p.o. for 4 weeks; T1D + CH100—diabetic rats treated with chrysin 100 mg/kg p.o. for 4 weeks. One-way analysis of variance (ANOVA) followed by Fisher’s LSD test was used for evaluation of the significance of the results. * *p* < 0.05, *** *p* < 0.001—in comparison with the healthy control rats. # *p* < 0.05—in comparison with the diabetic control rats (T1D group).

**Table 5:** Effects of chrysin administered for 4 weeks on histomorphometric parameters of the femoral diaphysis in rats with experimental T1D.

	Parameter/Group	Control	T1D	T1D + CH50	T1D + CH100
Femoral diaphysis	Tt.Ar (mm^2^)	9.543 ± 0.235	8.617 ± 0.272 *	8.465 ± 0.132 **	8.537 ± 0.340 **
	Ct.Ar (mm^2^)	5.927 ± 0.193	5.224 ± 0.116 **	5.228 ± 0.085 **	5.162 ± 0.191 **
	Ma.Ar (mm^2^)	3.616 ± 0.199	3.392 ± 0.175	3.237 ± 0.079	3.375 ± 0.215
	Ma.Ar/Tt.Ar (%)	37.8 ± 1.6	39.2 ± 0.9	38.2 ± 0.6	39.4 ± 1.4

The results are presented as means ± standard error of the mean (SEM). Control—healthy control rats; T1D—diabetic control rats; T1D + CH50—diabetic rats treated with chrysin 50 mg/kg p.o. for 4 weeks; T1D + CH100—diabetic rats treated with chrysin 100 mg/kg p.o. for 4 weeks. Tt.Ar—transverse cross-sectional area of the whole diaphysis; Ct.Ar—transverse cross-sectional area of the cortical bone; Ma.Ar—transverse cross-sectional area of the marrow cavity; Ma.Ar/Tt.Ar—transverse cross-sectional area of the marrow cavity/total diaphysis area ratio. One-way analysis of variance (ANOVA) followed by Fisher’s LSD test was used for evaluation of the significance of the results. * *p* < 0.05, ** *p* < 0.01—in comparison with the healthy control rats.

**Table 6:** Effects of chrysin administered for 4 weeks on histomorphometric parameters of the femoral metaphysis in rats with experimental T1D.

	Parameter/Group	Control	T1D	T1D + CH50	T1D + CH100
Femoral metaphysis	BV/TV (%)	33.05 ± 2.21	27.89 ± 4.55	27.80 ± 1.67	27.60 ± 1.12
	Tb.Th (μm)	47.34 ± 2.85	38.70 ± 5.40	41.04 ± 1.89	36.77 ± 2.01
	Tb.Sp (μm)	96.27 ± 4.58	102.02 ± 9.80	107.90 ± 5.94	96.54 ± 4.46
	Tb.N (1/mm)	6.98 ± 0.17	7.13 ± 0.25	6.76 ± 0.24	7.55 ± 0.32

The results are presented as means ± standard error of the mean (SEM). Control—healthy control rats; T1D—diabetic control rats; T1D + CH50—diabetic rats treated with chrysin 50 mg/kg p.o. for 4 weeks; T1D + CH100—diabetic rats treated with chrysin 100 mg/kg p.o. for 4 weeks. BV/TV—bone volume/tissue volume ratio; Tb.Th—trabecular thickness; Tb.Sp—trabecular separation; Tb.N—trabecular number. One-way analysis of variance (ANOVA) followed by Fisher’s LSD test was used for evaluation of the significance of the results.

**Table 7:** Effects of chrysin administered for 4 weeks on mechanical properties of the proximal tibial metaphysis in rats with experimental T1D.

Parameter/Group	Control	T1D	T1D + CH50	T1D + CH100
Displacement for yield point load (mm)	0.285 ± 0.062	0.217 ± 0.025	0.172 ± 0.032	0.155 ± 0.015
Maximum load (N)	65.0 ± 3.2	31.9 ± 2.3 ***	36.4 ± 3.5 ***	34.6 ± 5.3 ***
Displacement for maximum load (mm)	0.903 ± 0.060	0.531 ± 0.078 **	0.591 ± 0.088 **	0.694 ± 0.093
Energy for maximum load (mJ)	37.2 ± 3.0	10.7 ± 1.9 ***	13.8 ± 1.8 ***	16.3 ± 3.1 ***
Stress for maximum load (MPa)	46.0 ± 2.7	25.6 ± 2.1 ***	29.7 ± 1.9 ***	26.5 ± 2.6 ***
Fracture load (N)	48.7 ± 3.3	24.1 ± 2.5 ***	25.4 ± 2.4 ***	28.2 ± 5.3 ***
Displacement for fracture load (mm)	1.396 ± 0.065	0.911 ± 0.070 ***	1.050 ± 0.083 **	0.970 ± 0.082 ***
Energy for fracture load (mJ)	65.0 ± 3.7	21.4 ± 2.4 ***	28.0 ± 3.4 ***	24.8 ± 3.7 ***
Stress for fracture load (MPa)	34.8 ± 3.2	19.3 ± 2.1 ***	20.7 ± 1.5 ***	21.3 ± 2.7 ***

The results are presented as means ± standard error of the mean (SEM). Control—healthy control rats; T1D—diabetic control rats; T1D + CH50—diabetic rats treated with chrysin 50 mg/kg p.o. for 4 weeks; T1D + CH100—diabetic rats treated with chrysin 100 mg/kg p.o. for 4 weeks. One-way analysis of variance (ANOVA) followed by Fisher’s LSD test was used for evaluation of the significance of the results. ** *p* < 0.01, *** *p* < 0.001—in comparison with the healthy control rats (control group).

**Table 8:** Effects of chrysin administered for 4 weeks on mechanical properties of the femoral diaphysis and femoral neck in rats with experimental T1D.

	Parameter/Group	Control	T1D	T1D + CH50	T1D + CH100
Femoral diaphysis	Displacement for yield point load (mm)	0.275 ± 0.004	0.247 ± 0.017	0.260 ± 0.014	0.292 ± 0.014
	Maximum load (N)	139.2 ± 5.8	113.7 ± 6.4 **	121.1 ± 6.9 *	115.3 ± 4.3 **
	Displacement for maximum load (mm)	0.543 ± 0.034	0.497 ± 0.041	0.563 ± 0.033	0.533 ± 0.030
	Energy for maximum load (mJ)	45.2 ± 4.9	34.0 ± 5.6	40.2 ± 4.9	34.9 ± 3.6
	Stress for maximum load (MPa)	153.3 ± 4.4	150.0 ± 7.9	164.8 ± 5.9	155.0 ± 5.2
	Fracture load (N)	139.1 ± 5.7	113.1 ± 6.1 **	120.8 ± 6.9 *	113.0 ± 3.3 **
	Displacement for fracture load (mm)	0.548 ± 0.035	0.504 ± 0.045	0.569 ± 0.035	0.557 ± 0.045
	Energy for fracture load (mJ)	45.8 ± 5.0	35.0 ± 6.2	40.9 ± 5.1	37.8 ± 5.8
	Stress for fracture load (MPa)	153.1 ± 4.3	149.3 ± 7.7	164.4 ± 5.9	152.6 ± 6.6
	Maximum load in the femoral neck (N)	96.8 ± 4.2	84.8 ± 5.9	91.2 ± 3.8	88.2 ± 7.2

The results are presented as means ± standard error of the mean (SEM). Control—healthy control rats; T1D—diabetic control rats; T1D + CH50—diabetic rats treated with chrysin 50 mg/kg p.o. for 4 weeks; T1D + CH100—diabetic rats treated with chrysin 100 mg/kg p.o. for 4 weeks. One-way analysis of variance (ANOVA) followed by Fisher’s LSD test was used for evaluation of the significance of the results. * *p* < 0.05, ** *p* < 0.01—in comparison with the healthy control rats.

## Data Availability

Data are contained within the article.
